# Phase Quantification of Heterogeneous Surfaces Using DFT-Simulated Valence Band Photoemission Spectra

**DOI:** 10.1021/acsami.3c06638

**Published:** 2023-08-08

**Authors:** Roxy Lee, Raul Quesada-Cabrera, Joe Willis, Asif Iqbal, Ivan P. Parkin, David O. Scanlon, Robert G. Palgrave

**Affiliations:** † Department of Chemistry, UCL (University College London), 20 Gordon Street, London WC1H 0AJ, U.K.; ‡ Department of Chemistry, Institute of Environmental Studies and Natural Resources (i-UNAT, FEAM), Universidad de Las Palmas de Gran Canaria (ULPGC), Campus de Tafira, Las Palmas 35017, Spain; § Thomas Young Centre, UCL (University College London), Gower Street, London WC1E 6BT, U.K.; ∥ Diamond Light Source Ltd., Harwell Science and Innovation Campus, Didcot, Oxfordshire OX11 0DE, U.K.; ⊥ Materials Engineering, McGill University, 3610 University Street, Montreal, Quebec H3A 0C5, Canada

**Keywords:** XPS phase quantification, DFT, valence band, surface mapping, heterogeneous surfaces, polymorphs, photocatalysis, TiO_2_

## Abstract

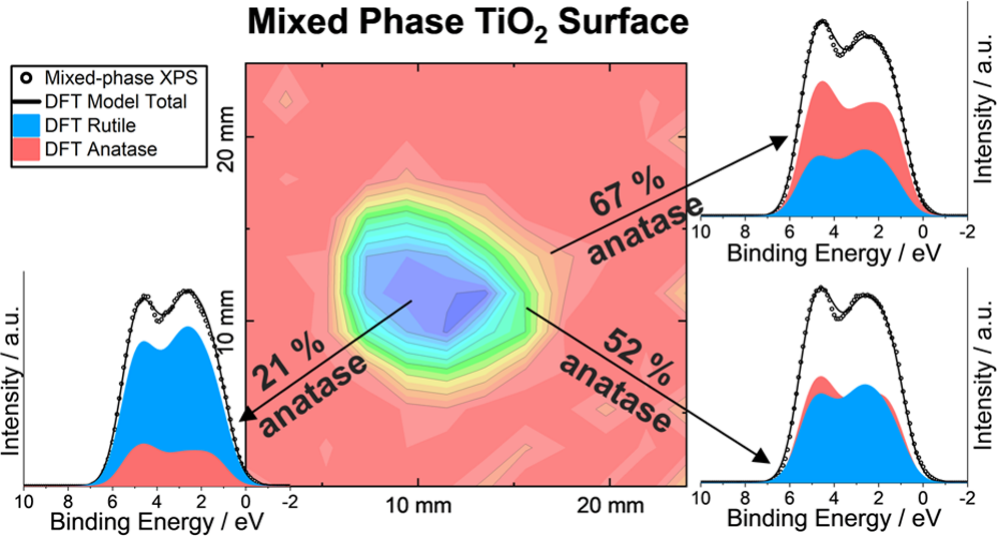

Quantifying the crystallographic phases present at a surface is an important challenge in fields such as functional materials and surface science. X-ray photoelectron spectroscopy (XPS) is routinely employed in surface characterization to identify and quantify chemical species through core line analysis. Valence band (VB) spectra contain characteristic but complex features that provide information on the electronic density of states (DoS) and thus can be understood theoretically using density functional theory (DFT). Here, we present a method of fitting experimental photoemission spectra with DFT models for quantitative analysis of heterogeneous systems, specifically mapping the anatase to rutile ratio across the surface of mixed-phase TiO_2_ thin films. The results were correlated with mapped photocatalytic activity measured using a resazurin-based smart ink. This method allows large-scale functional and surface composition mapping in heterogeneous systems and demonstrates the unique insights gained from DFT-simulated spectra on the electronic structure origins of complex VB spectral features.

## Introduction

Quantifying the concentrations of crystalline phases present in a sample has been a vital tool in chemistry, physics, geology, materials science, and related disciplines for over 80 years.^1^ The practice of quantitative phase determination is largely, but not exclusively, carried out using diffraction methods. In the well-known Rietveld method, an atomistic structural model for each phase is used to calculate diffraction intensities and angles, which are compared with the experiment, and the model, including parameters representing phase fraction, is then iteratively refined against the data.^2^ The high penetrating power of X-rays and neutron beams makes these diffraction techniques typically able to probe many micrometers or further into a sample, making the phase fractions obtained from refinement representative of the bulk material. However, for functional materials with applications dominated by surface processes, a major challenge lies in the quantification of crystalline phases at the surface. A wealth of information for the top ∼5 nm of a sample can be obtained using X-ray photoelectron spectroscopy (XPS), including identification and quantification of elemental surface composition, determination of oxidation state, and local chemical environment. XPS can therefore aid accurate characterization of the surface structure, which is of central importance in understanding reaction mechanisms and chemical processes in fields such as interfaces, nanotechnology, and heterogeneous catalysis, among others.

In XPS, core and valence electrons are ejected from the surface of a sample, giving rise to what can be considered two different types of spectra. Photoemission from core levels produces photoelectron peaks at characteristic binding energies, which are subject to chemical shifts depending on the local chemical environment. Determining the positions and intensities of these core lines is the basis of most routine materials analysis by XPS. Emission from core orbitals can be modeled using simple line shapes, commonly by a convolution of Gaussian and Lorentzian functions, and while final state effects can cause difficulties, in many cases, a straightforward interpretation of core line binding energy is possible. In contrast, the ejection of valence electrons results in significantly different spectra. By their nature, valence orbitals interact with neighboring atoms; they can no longer be assumed to be unaltered atomic orbitals, and so valence spectra have contributions from several orbitals and often show complex shapes influenced by the atomistic structure of the sample. Analysis of XPS valence band (VB) spectra is more challenging than core line analysis due to the complex nature of valence orbital contributions and interactions. Typical XPS analysis of the VB region band can reveal chemical changes induced by doping,^3−5^ defect and carrier behavior in semiconductors,^6−8^ or measure band offsets at a heterojunction.^9−12^ The work function of surfaces may also be measured by analysis of XPS valence bands, although this is more commonly carried out with ultraviolet photoemission spectroscopy (UPS).

Recently, it has been shown that structural information such as identification of crystalline phase,^13^ and quantification of phase fractions,^14−16^ can be extracted from the XPS VB spectrum of mixed-phase samples. By fitting the VB spectrum from a mixed-phase sample with experimental spectra measured from each of the pure phases, the amount of each phase can be determined. Although this extraction of structural information from XPS has so far been demonstrated only for TiO_2_ and FeO*_x_*, these examples show that quantitative information can be obtained from the VB region by fitting the spectrum with empirically derived models. Far greater potential, however, lies in the possibility of fitting with a simulated spectrum derived from an atomistic model and then refining the model to optimize the fit, mirroring the Rietveld method in powder X-ray and neutron diffraction.

Density functional theory (DFT) is a suitable theoretical framework for producing such simulations. Experimental VB spectra provide information about the density of states (DoS) within a sample and so may be, with caution, comparable to theoretical DoS calculated with DFT. As noted by Bagheri et al.,^17^ the orbital energies in DFT do not correspond to ionization potentials, and photoemission spectra contain several other important contributions apart from loss-free electron ionization; nevertheless, a simulated DFT spectrum, produced from a DoS suitably broadened and weighted with photoionization cross-sections, can match very closely to experimental XPS VB spectra.^8,18−20^ Comparison of experimental and simulated valence band spectra has been used, for example, to investigate energy-dependent VB spectra for several oxides,^17^ determine the valence band maximum (VBM) in metal halide perovskites^21−23^ and metal nitrides,^24^ probe the valence states in cerium vanadate-based materials,^25^ identify the electronic structure origins of poor performance in tin monosulfide (SnS) solar cells,^11^ understand changes to the VB spectrum seen on the varying incident photon energy,^26^ explore the effect of polymorphism on the electronic structure of Ga_2_O_3_ phases,^27^ and study lifetime and satellite effects in tungsten metal.^28^

Despite this wide range of uses, combined VB XPS and DFT studies predominantly involve qualitative comparison of experimental and simulated spectra. A simulated spectrum is typically judged by eye to be a good or poor match with the experiment and relevant conclusions are drawn. Advances in both computational methods, allowing more accurate simulation of valence electronic structure, and XPS instrumentation, allowing far more rapid collection of the low-intensity valence band region, have created the potential for advancement in this area. Here, we undertake a new approach: the least-squares fitting of experimental valence band spectra using DFT-simulated spectra for quantitative analysis. We apply this approach to mapping the anatase/rutile composition across the surface of mixed-phase titania (TiO_2_) thin films. Furthermore, taking advantage of the different photocatalytic behavior of the two TiO_2_ polymorphs, the composition mapping obtained from VB analysis was compared with the photocatalysis rate, thus allowing us to directly probe the surface phase fraction and relate this to the rate of heterogeneous catalysis.

## Experimental and Computational Methods

### Sample Preparation

All chemicals were purchased from Sigma-Aldrich. Four anatase TiO_2_ thin films were deposited on quartz substrates (25 × 25 mm^2^) using atmospheric-pressure chemical vapor deposition (APCVD). The synthesis was carried out using titanium isopropoxide as a single-source precursor. The precursor was initially contained in a stainless-steel bubbler at 150 °C and subsequently carried (precursor gas flow, 1.5 L min^–1^) under preheated nitrogen carrier gas through a mixing chamber heated to 200 °C into a cold-wall reactor. The CVD reactor consisted of a quartz tube with a 320 mm-long graphite block with three inserted Whatman heaters. The total gas flow in the system was 10 L min^–1^. All components of the CVD apparatus were kept at 150 °C, and temperature control of the individual components was monitored using Pt–Rh thermocouples.

The initial deposition of anatase TiO_2_ coatings was carried out at a substrate temperature of 500 °C for 2 min. This procedure resulted in the formation of an anatase TiO_2_ film, with no rutile phase detected by thin-film XRD or Raman spectroscopy. One of these films was retained with no further treatment as a pristine anatase reference. Two of the films were subsequently treated under oxy-propane flame annealing localized at the corner and center regions of the films in order to induce the local formation of rutile through thermally induced phase transformation. These samples are herein referred to as r-corner and r-center samples, respectively. The temperature in the flame-annealed area was estimated using a Mikron 9104 IR camera, ranging between *T* = 1000–1200 °C. A third sample was annealed in a furnace at 1000 °C for 5 h to allow for a complete transformation into a pure rutile phase. Both pristine anatase and the rutile films produced by furnace annealing were used as reference samples.

### X-ray Photoelectron Spectroscopy

XPS was carried out using a Thermo K-alpha spectrometer equipped with a monochromated Al Kα X-ray source (1486.6 eV) in constant analyzer energy mode, with an analyzer mean radius of 125 mm. No special steps were taken to clean the samples prior to measurement, and no etching was carried out on the samples as this is known to cause a reduction of TiO_2_. Sample charging was prevented by the use of a dual-beam flood gun. A pass energy of 50 eV was used to record high-resolution valence band spectra for the TiO_2_ thin-film samples detailed above. For the anatase and rutile samples, VB spectra were recorded at four points and averaged to produce representative VB spectra for each polymorph. The analysis area at each point was 400 μm in diameter. For the mixed-phase samples, spectra were recorded for a square grid of 12 × 12 points, which were spaced 2.1 mm apart.

Experimental valence band spectra were processed as follows using CasaXPS:^29^ the data were smoothed using a 3-point moving average filter, and the valence band maximum (VBM) was aligned to 0 eV by fitting the low binding energy edge with a complementary error function. This function is chosen to provide a consistent method for determining the edge; other choices would lead to slightly different offsets of the spectra but would not impact any other conclusions. The background is an intrinsic feature of experimental spectra, which arises from the inelastic scattering of photoelectrons, and is modeled and subtracted for comparisons with simulated spectra. The Shirley background was originally proposed for the comparison of XPS VB spectra with broadened theoretical band structure calculations^30^ and is used here. The inelastic background is modeled at a given energy, *E*_b_(*x*), by the integrated area between the intensity at *E*_b_(*x*) and the lower binding energy limit. Although Shirley did not make the physical meaning of this background model clear, good agreement has been found between the experiment and theory, and it remains one of the most popular methods used to date.

### Raman Spectroscopy

Raman spectra were acquired using a Renishaw micro-Raman spectrometer with a sensitive CCD detector coupled to a microscope for point-by-point analyses. Spectra were measured using an incident wavelength of 514 nm with an exposure time of 3 × 60 s. The spectra for horizontal linear cross-sections of all samples were recorded for a set of 12 analysis points spaced 2.1 mm along a diagonal of the substrate to include the rutile-rich corner region with analysis points. Phase quantification via Raman spectroscopy was achieved using a methodology for determining the concentration of anatase or rutile in mixed-phase TiO_2_ systems.^31^ The analysis region was confined to 310–750 cm^–1^, with five individual phonon modes, 3 anatase, and 2 rutile, fitted with Lorentzian functions using the OriginLab platform.

### Photoactivity Mapping

Local photocatalytic activities were assessed using a standard test following the photoreduction of a resazurin-based (smart) ink.^32,33^ The ink was prepared from a 1.5% hydroxyethyl cellulose (HEC) solution that was cooled to 2 °C overnight prior to further use. The rest of the components in the ink (given per 10 g of HEC) were subsequently added under strong stirring conditions, in order: 1 g of glycerol (30 min stirring time), 10 mg of resazurin dye (2 h stirring time), and 20 mg of polysorbate (30 min stirring time). The stock dye mixture was then stored at 2 °C and again stirred for 30 min prior to each use. The ink was coated onto the thin-film samples by spin coating. The samples were irradiated under a blacklight-bulb (BLB) UVA lamp (Vilber-Lourmat, 2 × 8 W, λ = 365 nm). The irradiance of the lamps at the sample point was measured using a UVX radiometer (UVP) as *I* = 2 mW cm^–2^. Color mapping was traced using RGB analysis (12 × 12 pixel grid) in MATLAB software (Supporting Information).

### Computational Methods

The initial stage in the simulation of the VB spectra involved the theoretical determination of the ground state electronic structure using density functional theory (DFT). The periodic DFT code Vienna Ab initio Simulation Package (VASP)^34,35^ was employed for the calculations, which uses a plane-wave basis set to describe the valence electronic states. Lattice parameters were sourced through the ICSD database, geometry optimization and electronic density of states (DoS) calculations were performed using the generalized gradient approximation (GGA), implemented in the Perdew–Burke–Ernzerhof (PBE)^36^ functional adapted for solids (PBEsol).^37^ The plane-wave cut-off energy and *k*-point density were checked for convergence for each system to within 1 meV/atom. For geometry optimization, a plane-wave cut-off of 600 eV was implemented, and a Γ-centered grid with *k*-point densities of 6 × 6 x 3 for the anatase phase and 5 × 5 x 8 for the rutile phase. For the DoS calculations, *k*-point densities of 8 × 8 x 3 and 5 × 5 x 8 were used for anatase and rutile, respectively.

### Valence Band XPS/DFT Refinement Method

To produce simulated Al Kα XPS VB spectra from the DFT calculated partial density of states (pDoS), the contributions from each orbital must be weighted by energy-dependent photoionization cross-sections, spectral broadening must be applied, and an offset must be applied to bring the DFT and binding energy scales into alignment. The latter of these is relatively straightforward, but the former two parameters need special care, and for both the broadening and weighting of orbital contributions, there is no consistent approach taken by previous researchers in the literature. In this section, we briefly review the different approaches taken in the literature, first, to spectral broadening and, second, to the weighting of orbital contributions. Finally, we present the methods used to produce simulated XPS spectra in the current work.

Spectral broadening is universally carried out using a Gaussian component to simulate X-ray linewidth, spectrometer resolution, and vibrational broadening, and a Lorentzian component is applied to simulate lifetime effects.^25,38−42^ Beyond this, approaches to the spectral broadening operation can be divided into two main categories: first, those where the broadening applied is based on a measured energy resolution of the spectrometer, measured either by the fitting of the Fermi edge of a metal or by measuring the width of a core line of a well-prepared standard, e.g., Ag metal. Second is the approach of empirically determining the broadening to give the best fit to the experimental data. Examples of the former case are early work by Wolfram and Ellialtioğlu,^43^ who used the spectrometer resolution to broaden calculated oxide perovskite DoS, and a more recent study by Parvizian et al., who similarly broadened their calculated DoS of Cu_3_N.^24^ In both these cases, the broadened calculated spectrum is considerably narrower than the experimental XP spectrum.

Strict use of spectrometer energy resolution is an attractive approach but has some difficulties. In many cases, the Lorentzian portion is empirically fitted or estimated, for example, using the range of lifetime broadenings suggested by Fadley and Shirley (0.1–1.0 eV).^44^ Some researchers have applied a variable Lorentzian lifetime broadening with *E*^2^ dependence to simulate the changing lifetime across the VB region.^25,45^ The use of purely empirical broadening, with no relation to a measured energy resolution, is exemplified by Bagheri and Blaha, who used a range of Gaussian broadenings from 1.05 to 0.36 eV (with a fixed Lorentzian broadening of 0.1 eV) to fit VB spectra of a range of oxides.^17^ There are several other examples of empirically determined broadening producing well-matching simulated spectra, which can require convolution of the pDoS with a Gaussian of larger width than the instrumental resolution.^23,43,46−48^ The necessity for a larger broadening of the VB compared with instrument resolution may point to an additional contribution to the VB width; for example, the vibrational broadening of the VB may be larger than that seen in core lines.

In the present work, we implemented a least-squares refinement method to fit the DFT-simulated spectra to experimental XPS spectra empirically by minimizing the residual sum of squares RSS (eq S1). This allowed us to obtain broadening parameters that would then be used for the mixed-phase fitting. Values of 1.3 and 0.1 eV for Gaussian and Lorentzian contributions, respectively, were determined to give the best fit to experimental data. These are considerably larger than our measured instrument energy resolution, either using the Ag 3d_5/2_ FWHM taken from a clean silver foil (0.50 eV) or by measuring the Fermi edge of gold (0.42 eV), meaning that we replicate the literature results discussed above, that broadening considerably wider than the standard resolution is needed to fit VBs well with DFT-derived spectra. After broadening, a zero-point binding energy shift was refined to align simulated and experimental spectra.

Turning to the method of weighting each orbital contribution in the pDoS by energy-dependent tabulated photoionization cross-section values, this process is not straightforward as there are different values available for Al Kα X-ray photoionization (photon energy 1486.6 eV), such as those by Scofield,^49^ and from Yeh and Lindau (referred to as YL cross-sections).^50^ In addition, there are no available cross-sections for energy levels unoccupied in the neutral atomic species; therefore, the Ti 4p photoionization cross-section must be estimated. The approach taken by Mudd et al. to estimate the unoccupied Cd 5p orbital contribution in CdO was to apply the ratio of the cross-sections of the In 5p and In 5s orbitals to the Cd 5s cross-section value,^18^ as this is the next element with an occupied 5p orbital. Bagheri et al. extrapolated the cross-section values for neighboring atoms to obtain estimates for empty subshell cross-section values.^17^ In the case of PbO_2_, Bagheri et al. estimated the 6d cross-section parameter by extrapolating from neighboring elements that contain occupied 6d states in the free atom (Ac, Th, Pa), and in the case of ZnO, they extrapolate the 4p value from Ga and Ge. An alternative method is to use tabulated values for filled states and to estimate the contribution from empty states empirically by fitting the model to experimental data.^28,51^ This approach has been used recently by Kalha et al. to simulate the VB spectrum of tungsten metal with an optimized weighting for W 6p, with a reasonable fit also obtained from the tabulated cross-section for W 5p.^28,51^

The correction of pDoS using tabulated cross-sections was described as “essential” in an experimental and theoretical investigation of the electronic structure of CdO by Dou et al.,^52^ who use YL cross-sections. In contrast, King et al. found good agreement between experimental spectra and DoS calculations for CdO, ZnO, and MgO and stated that there is no need for cross-section correction as the anion p and cation s cross-sections are similar.^53^ Walsh et al. also reported that the experimental curve for α-Bi_2_O_3_ is well represented by the DoS and concluded that the cross-sections for O 2p, Bi 6s, and Bi 6p are very similar,^54^ which is not in accordance with the YL tabulated values. A study on the electronic structure of lanthanide scandates by Mizzi et al. reported that tabulated cross-section values are unreliable for the valence band; therefore, they use the literature values as starting points and then vary them.^55^ Similarly, a study on rutile TiO_2_ by Woicik et al. approximated the Ti 4p cross-section as equal to that of Ti 4s states and then adjusted all pDoS weightings to obtain good agreement with site-specific VB XPS spectra.^56^ Clearly, the suitability of tabulated cross-section values for simulating XPS spectra remains a point of contention in the field.

To establish which tabulated photoionization cross-section values to use and an appropriate estimate for the Ti 4p cross-section, we explored different methods of orbital weighting. Both Scofield and YL cross-sections were evaluated and implemented using the software package Galore.^39^ We compared weighting the Ti p states with the Ti 3p cross-section to an estimated Ti 4p value by multiplying the Ti 4s value by the 4p/4s ratio of a neighboring atom (full details are in the Supporting Information.) Briefly, the Scofield-derived simulated spectra gave very poor agreement with the experimental VB, regardless of the estimate for the Ti 4p value. The YL-derived simulated spectra presented improved fits, with the estimated 4p value offering a reduction in the **RSS** compared to the 3p value for both polymorphs. We also calculated optimized weightings by starting with the YL cross-sections and the 4p estimate and then refined the contributions with both polymorphs and took an average for our final values. Notably, the refined values indicate an enhanced contribution from both Ti and O s and p orbitals. Increased contribution from s and p orbitals has been found to give better agreement in previous joint experimental and theoretical photoemission studies,^46,56−59^ and deviations from atomic cross-sections may be understood as arising from solid-state effects. All theoretical spectra presented herein have been simulated using optimized parameters obtained from the XPS/DFT refinement method described here.

## Results and Discussion

**Figure 1:**
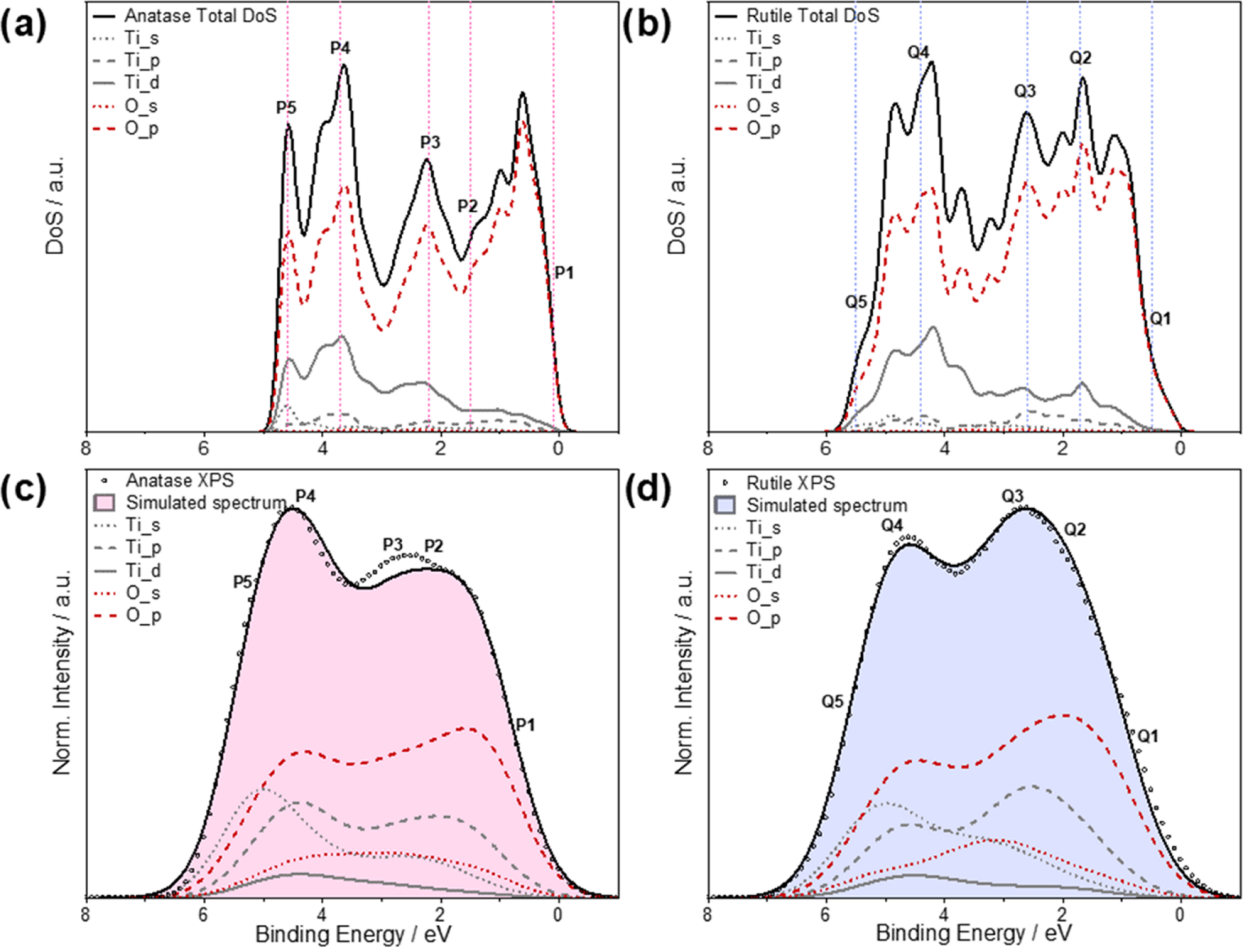
Partial DoS (pDoS) for (a) anatase and (b) rutile phases of TiO_2_, calculated within the DFT framework. A small degree of Gaussian broadening (0.1 eV) was applied to help visualize the data. The simulated VB spectra of (c) anatase and (d) rutile decomposed into their different angular momentum components and scaled to equal heights of the overlaid XPS spectra. The simulated spectra were derived from the pDoS by correction with several refined parameters (see Experimental and Computaional Methods section) photoionization cross-section values and Gaussian (1.3 eV) and Lorentzian (0.1 eV) broadening. The experimental spectra were smoothed using a 3-point moving average filter and have been corrected via Shirley background subtraction. The experimental valence band maximum has been aligned to 0 eV, and the simulated spectra have been aligned with the experimental using a least-squares fitting method. The regions labeled **P1–5** and **Q1–5** indicate regions of interest and are discussed in the text. The positions relative to the DFT energy scale are: **P1**, 0.1; **P2**, 1.5; P**3**, 2.2; **P4**, 3.7; **P5**, 4.6 eV and rutile: **Q1**, 0.5; **Q2**, 1.7; Q**3**, 2.6; **Q4**, 4.4; and **Q5**, 5.5 eV.

The DFT calculated partial density of states (pDoS) for both anatase and rutile TiO_2_ is dominated by the O p states in the valence band region (Figure 1a,b). This is consistent with other *ab initio* electronic structure calculations^25,38−42^ and supports a model of TiO_2_ as a predominately ionic compound. However, there is also a considerable contribution from Ti d states that have non-zero overlap integrals with the O p states, supporting a degree of hybridization between metal and oxygen states.^56,60,61^ There are also non-zero contributions from Ti s, Ti p, and O s states. Energy dependence of photoionization cross-sections means that the VB spectrum of both polymorphs appears differently under different excitation wavelengths. Henceforth we shall only refer to the Al Kα excited VB spectra (photon energy 1486.6 eV).

To produce the simulated spectra, the pDoS were corrected by several refined parameters obtained using the method described in the previous section. After applying the refined spectral broadening, photoionization cross-section, and binding energy shift parameters, the characteristic features of the XPS VB spectra of both anatase and rutile are well represented (Figure 1c,d). The experimental spectrum for each polymorph has a distinctive shape in the binding energy range 0–10 eV, which is well established in the literature and is consistently observed in single crystal, epitaxial thin film, and polycrystalline samples.^15,62−66^ Both polymorphs show a VB feature that is very similar in width, around 6.5 eV, with two distinct local maxima separated by approx. 2.5 eV. In the case of anatase, the higher binding energy maximum has greater intensity, whereas, in rutile, the lower binding energy maximum has greater intensity.

The features of the O p and Ti d components in the simulated spectra are similar for both polymorphs and have previously been interpreted in terms of the molecular-orbital (MO) bonding model for a first-row transition metal in an octahedral field.^56,61,67^ The most stable σ bonding interactions arise from the hybridization of the O p_σ_ and Ti e_g_ orbitals and constitute the higher binding energy feature in the O p and Ti d spectra. At lower binding energies, there are contributions from π bonds between O p_π_ and Ti t_2g_ states, and finally, the lowest binding energy region of the VB is predominantly non-bonding O p_π_ in nature.

**Figure 2:**
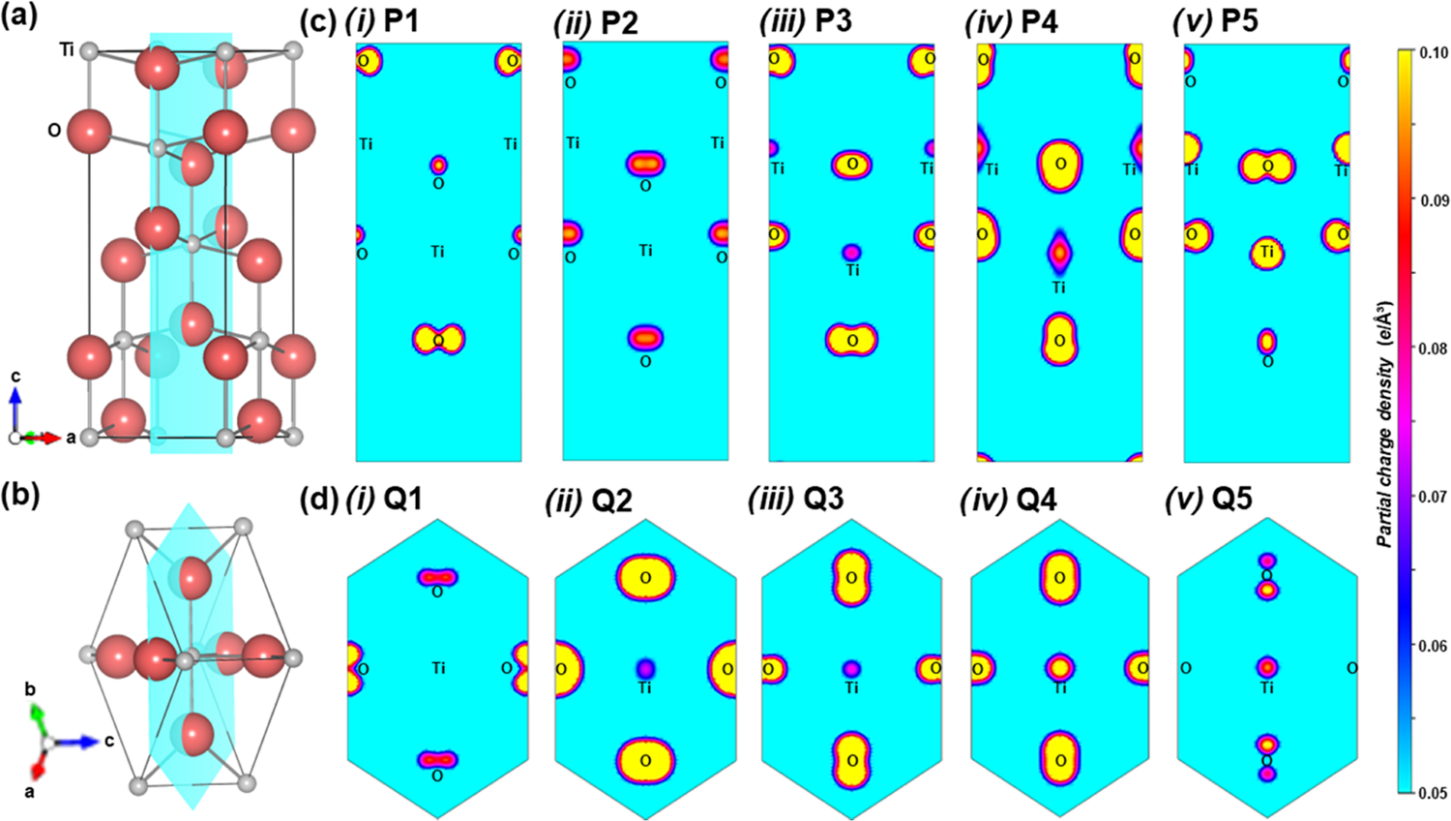
Contributions to the electron density at characteristic features in the VB spectra can be visualized by contour plots of the partial charge density. Lattice planes cutting through the central TiO_6_ octahedra in the unit cell are shown for (a) anatase and (b) rutile, which bisects the central Ti atom (gray spheres), both axial and two equatorial oxygen atoms (red spheres). These were used to obtain partial charge densities for states between selected small energy intervals in (c) anatase: (i) P1, (ii) P2, (iii) P3, (iv) P4, and (v) P5 and in (d) rutile: (i) Q1, (ii) Q2, (iii) Q3, (iv) Q4, and (v) Q5. The range of isosurface values is set to 0.05–0.10 e/Å^3^ in each plot.

To further investigate features of interest within the VB spectra, we compared the partial charge densities across planes that bisect the central TiO_6_ octahedra (Figure 2) for small energy intervals in the DFT calculations, which correspond to the regions labeled as **P1–P5** and **Q1–Q5** in Figure 1. In agreement with the MO bonding scheme, states sampled close to the VBM (**P1** and **Q1**) are predominantly O p in character for both polymorphs, with no electron density on the Ti sites for the set range of isosurface values (0.05–0.10 e/Å^3^). At intermediate energies, π bonding characteristics are displayed in both polymorphs by states **P3** and **Q2** and toward the lower energy (higher BE) edge, states **P4, P5** and **Q4, Q5** display σ bonding interactions. Differences between the anatase and rutile partial charge densities are presented by states sampled near the characteristic spectral maxima. States **Q2** and **Q3** show bonding interactions that contribute to the principal maximum in the rutile spectra, whereas this is much weaker for the states **P2** and **P3** in anatase, with **P2** being essentially O p in character. Similarly, states sampled close to the higher BE edge, **P5** and **Q5**, exhibit higher partial charge densities in anatase. These states contribute to the more intense high BE feature in anatase and the shallower high BE tail in rutile that are mirrored in the XPS spectra.

**Figure 3:**
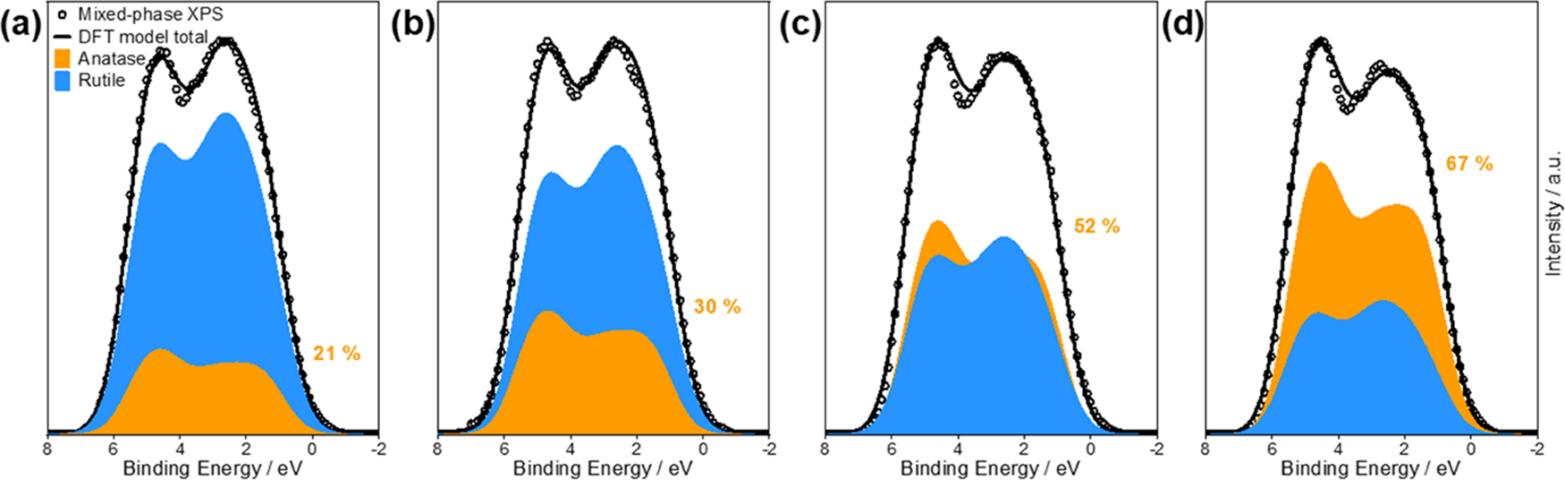
Mixed-phase fitting models based on DFT-simulated spectra applied to VB XPS spectra taken from 5 points of mixed-phase composition from the r-center sample. Each graph depicts the ratio of each phase that generates the lowest residual sum of squares (RSS) with the phase fraction of anatase: (a) 21%, (b) 30%, (c) 52%, and (d) 67%.

The characteristic shapes of the anatase and rutile VB spectra can be used to quantitatively determine phase composition using VB XPS spectra recorded on mixed-phase TiO_2_, which has previously been achieved using peak models that hold no physical basis.^14,15^ We present a new method of surface phase quantification here, using the DFT-simulated spectrum of each polymorph to develop mixed-phase fitting models. The simulated mixed-phase spectra were calculated by a linear combination of anatase and rutile simulated spectra, which were fitted to VB spectra recorded from a 12 × 12 grid of points across the mixed-phase r-corner and r-center films, each point around 2 mm apart. During fitting, the binding energy positions of each component were allowed to vary freely, their intensity ratios were allowed to vary between 0 and 100%, and a scale factor was applied such that the model total was equal to the maximum intensity of the experimental spectra. The anatase:rutile ratio with the minimum residual sum of squares (RSS) was used to estimate the surface phase fraction at that point. The resulting fits from applying the models to 5 points on the r-center sample are shown in Figure 3. This was compared to an empirical fitting model using anatase and rutile XPS VB spectra measured from the reference films, which gave similar estimates for the composition (Supporting Information).

**Figure 4:**
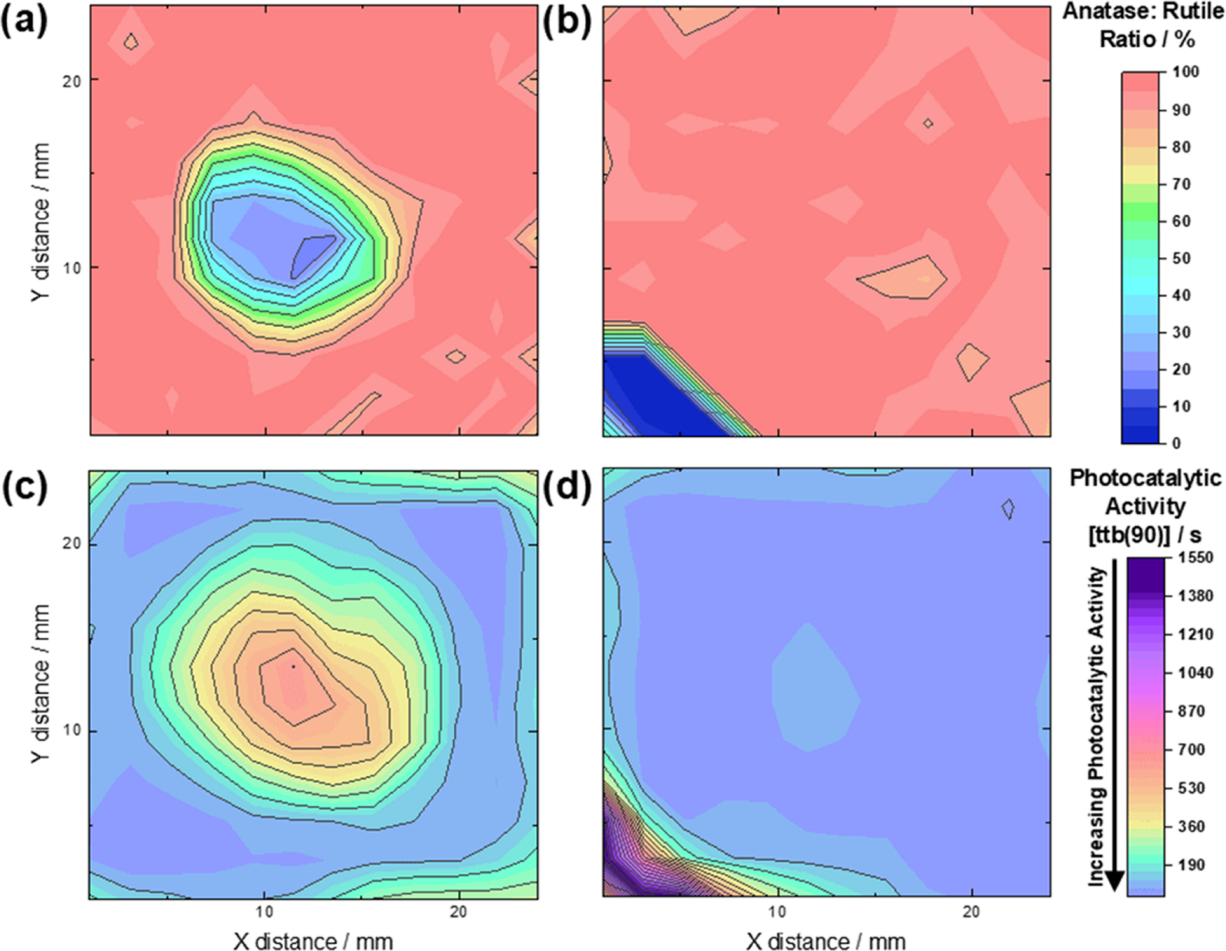
(a,b) Composition maps showing the anatase:rutile surface phase fraction across mixed-phase TiO_2_ samples as determined using a fitting model derived from DFT-simulated spectra. (c, d) The DFT fitting model successfully identifies the rutile-rich regions, which are evidenced by photocatalytic activity maps following the reduction of resazurin ink. The time to bleach ttb(90) is the time taken for 90% of the transformation in ink to occur in seconds.

**Figure 5:**
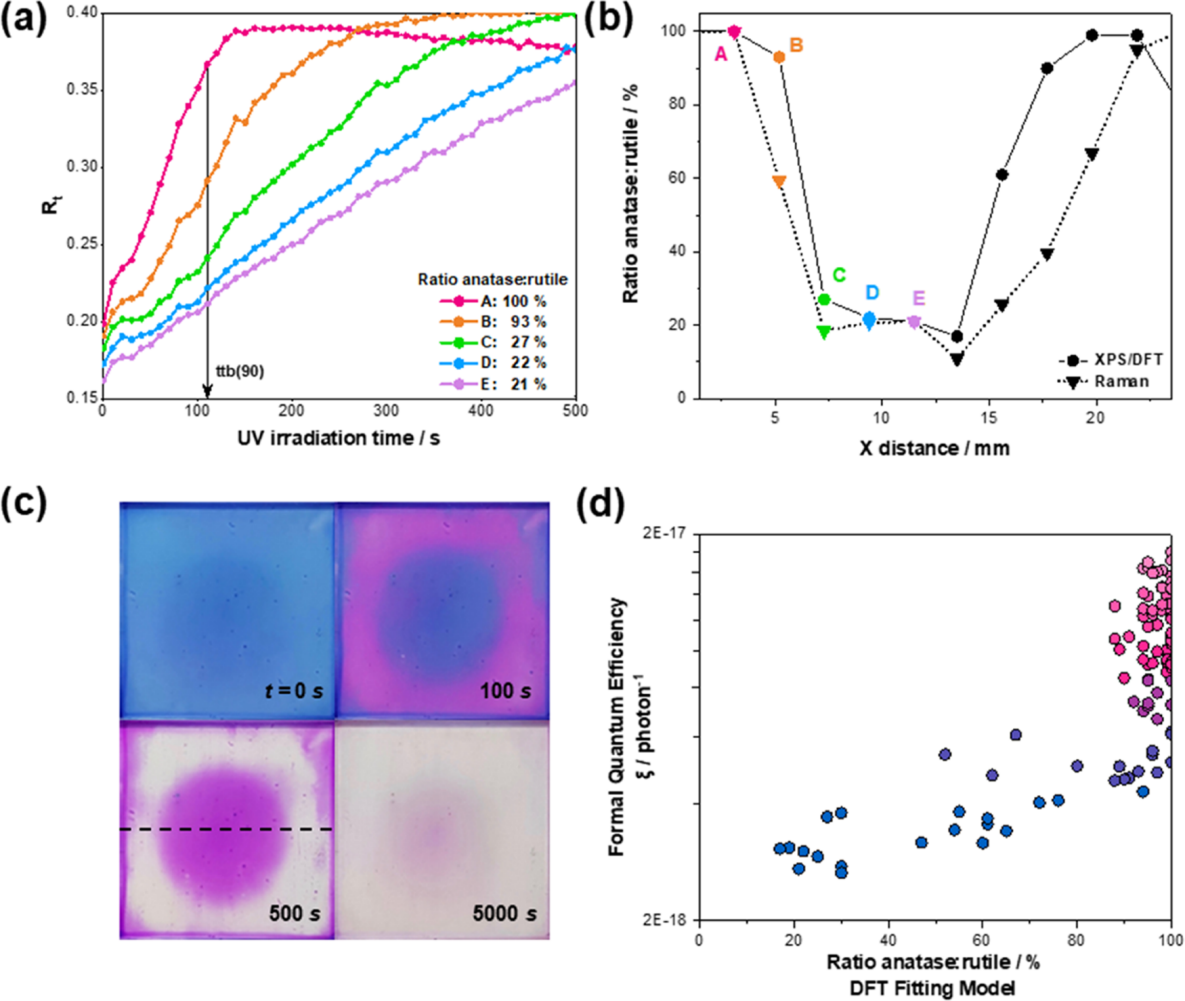
(a) Normalized red components (*R*_t_) of the resazurin dye upon irradiation time over the r-center sample. (b) The corresponding percentages of surface anatase as determined by the DFT fitting model are indicated and refer to the points labeled (A–E). (b) The compositions are estimated by the DFT fitting model and by Raman analysis for a linear cross-section of the r-center film, which is indicated by the dotted line in (c). (c) Photographic images of the dye-coated r-center film after exposure to UV irradiation showing the slower transformation of the resazurin dye in the rutile-rich center. Irradiation time in seconds is indicated for each photograph. (d) Variation of local formal quantum efficiencies, ξ (units, photon^–1^), defined here as the *R*_z_ photoreduction rate of per incident photon, with the local phase fraction of anatase predicted by the DFT-simulated fitting model. The ξ values are plotted on a log scale. Color mapping of the points is based on the anatase:rutile ratio.

Mapping the compositions estimated from the DFT fitting model allowed for clear identification of the respective surface rutile-rich regions in the r-center and r-corner samples (Figure 4a,b). The rutile content reached around 80% in the center of the r-center sample, and 100% rutile was observed in the corner of the r-corner sample. Structural analysis was also carried out using micro-Raman spectroscopy to establish a comparison with the XPS/DFT composition mapping. Details of the Raman studies are given in the Supporting Information. The reference samples were determined by Raman spectroscopy to be 100% anatase and rutile. Figure 5b shows a comparison of the cross-section of the r-center film, analyzed by both XPS/DFT and Raman spectroscopy. Both techniques indicate that the center of the film is ca. 80% rutile, although Raman spectroscopy finds a slightly wider region of rutile in the r-center film. This difference may be due to the relative surface sensitivity of the two methods; the transparent nature of the films to the Raman excitation wavelength used here means that the expected Raman analysis depth is large compared to XPS probing depth, which is estimated at 5 nm.

We studied the functional properties of the mixed-phase surfaces by measuring the photocatalytic activity using an indicator (or smart) ink.^32,68,69^ The smart ink test is based on the rate of color change of the resazurin dye.^33^ Details of this method are provided in the Supporting Information. Briefly, in the presence of an underlying photocatalytic coating under irradiation, resazurin (*R*_z_) is reduced to resorufin (*R*_f_), which is evidenced by a color change from blue to pink. *R*_f_ may then be destroyed in a further reduction step, leading to colorless products. The two TiO_2_ polymorphs show different photocatalytic behaviors; anatase is an efficient photocatalytic material, while rutile is inactive in the transformation of the *R*_z_ dye (Figure S6). This is evidenced by the inspection of Figure 5c, as the rutile-rich region (consisting of up to around 80% rutile from XPS-DFT analysis) in the r-center sample takes much longer to transform. Photographic analysis of the films upon irradiation provided average RGB values (given as normalized red component, *R*_t_) across a 12 × 12-point grid matching with the XPS analysis grid. An example of this analysis is shown in Figure S7. A plot of *R*_t_ values as a function of irradiation time thus allowed the determination of the time-to-bleach taken for 90% (ttb(90)) of the transformation of the ink for different anatase/rutile ratios (Figure 5a). Short ttb(90) values correspond to high photocatalytic activity, and these are seen in regions of high anatase content, gradually increasing with increasing rutile content at the film surface. Mapping of ttb(90) values was carried out using the mapping coordinates established in the XPS analysis. As an alternative measure of photocatalytic performance, we defined formal quantum efficiencies, ξ (units, photon^–1^), based on *R*_*z*_ photoreduction rates per incident photon (for methodology, see the Supporting Information). This allowed a correlation between theoretical phase fraction and experimentally determined photocatalytic activity (Figure 5d). For both measures of photocatalysis, i.e., the ttb(90) and quantum efficiency, the anatase-rich regions performed significantly better than rutile-rich regions. Figure 5d shows that the formal quantum efficiency increases exponentially with increasing anatase content, up to around 90% anatase (note the log scale on the *y*-axis in Figure 5d). In regions with anatase surface content above 90%, the quantum efficiency is much higher but does not have a simple trend with phase fraction. Here, the precise spatial arrangement of the phases may be a contributing factor, something that might be probed by angle-resolved XPS or hard X-ray photoemission spectroscopy (HAXPES), but this is beyond the scope of this work. Overall, the XPD-DFT phase fraction correlates well with photocatalytic activity.

The methodology proposed here is applied to a well-known photocatalyst but might be extended to many other material systems. It was advantageous here to analyze an anatase–rutile mix, where both individual components could be produced separately and their spectra measured. With a better understanding of how to produce simulated spectra from the DFT electronic structure, the method might be used where isolation of the individual phases is not possible. Additionally, parameters of the DFT simulation other than phase fraction might be refined, for example, defect concentration, dopant level, surface reconstruction, and crystal strain. To accomplish this, a greater understanding of spectral simulation from DFT electronic structure calculation is required. We hope the demonstration of this first step in quantitative refinement may encourage further work in this area.

## Conclusions

A new method of surface structural mapping was demonstrated using DFT-fitted valence band XPS analysis, applied to anatase/rutile mixed-phase TiO_2_ films. Different anatase:rutile ratios were obtained after flame annealing of anatase films from chemical vapor deposition. Theoretical Ti and O partial density of states from DFT were decomposed into their angular momentum components, and the electronic structure was interpreted within a σ and π bonding scheme. Significant differences between XPS spectral maxima were found to originate from high partial charge densities near the higher VB edge in anatase and increased Ti p and Ti d electron density near the lower BE maximum in rutile.

Mixed-phase fitting models were developed using DFT-simulated spectra and compared to an empirical fitting model using VB XPS recorded from pristine anatase and rutile films. The models were applied to mapping the surface phase fraction on the mixed-phase films, with clear identification of the rutile-rich regions in each case. The spatially resolved photocatalytic activity was measured using photocatalytic reduction of a resazurin-based smart ink, which highlighted the same rutile-rich areas as evidenced by XPS and DFT analysis. Calculation of local formal quantum efficiencies allowed a correlation between the theoretical phase fraction and experimentally determined photocatalytic activity. The concept of refinement of a DFT-derived model against experimental spectroscopic data may have many further applications beyond phase quantification, for example, refinement of structural parameters, defect concentrations, or surface reconstructions and orientations.

## Supporting Information Available

XPS data (ZIP)
Details of photoionization cross-section refinement and additional photocatalysis data (PDF)
