# Supplementary material for: Phase Quantification of Heterogeneous Surfaces Using DFT-Simulated Valence Band Photoemission Spectra
Source: ACS Appl Mater Interfaces. 2023 Aug 8;15(33):39956–65. doi: 10.1021/acsami.3c06638 (PMC10450682; doi:10.1021/acsami.3c06638)
Supplement: Supplementary file 2 — am3c06638_si_002.pdf [file am3c06638_si_002.pdf]

Supporting Information

Phase quantification of heterogeneous surfaces using DFT simulated valence band photoemission spectra

Roxy Lee[a], Raul Quesada-Cabrera[a,b]*, Joe Willis[a,c,d], Asif Iqbal[e], Ivan Parkin[a], David O. Scanlon[a,c], Robert G. Palgrave[a]*

[a] Department of Chemistry, UCL (University College London), 20 Gordon Street, London WC1H 0AJ, UK
[b] Department of Chemistry, Institute of Environmental Studies and Natural Resources (i-UNAT, FEAM), Universidad de Las Palmas de Gran Canaria (ULPGC), Campus de Tafira 35017, Spain
[c] Thomas Young Centre, UCL (University College London), Gower Street, London, WC1E 6BT, UK
[d] Diamond Light Source Ltd., Harwell Science and Innovation Campus, Didcot, Oxfordshire, OX11 0DE, UK
[e] Materials Engineering, McGill University, 3610 University Street, Montreal QC H3A 0C5, Canada

Email : r.palgrave@ucl.ac.uk; raul.quesada@ulpgc.es

Least-squares Fitting Methods

A least-squares refinement method was implemented to fit the XPS spectra with model spectra, whereby optimal parameters were obtained by minimising the residual sum of squares $\boldsymbol{RSS}$ (**Eq. S1**) between the observed and calculated spectra.

$$\boldsymbol{RSS} = \sum_i (\boldsymbol{I}_i^{obs} - \boldsymbol{I}_i^{calc})^2 \qquad \textbf{Eq. S1}$$

Where $\boldsymbol{I}_i^{obs}$ and $\boldsymbol{I}_i^{calc}$ are the intensities observed and calculated in the spectra, respectively.

Refinement of Photoionization Cross-section Weightings

The simulated spectra from different methods of cross-section correction were compared and evaluated according to the least-squares fitting method described above. The photoionization cross-section values applied are shown in **Table 1** and **Table 2**, and corresponding $\boldsymbol{RSS}$ values are indicated in **Table 3**. The corresponding simulated spectra are shown in **Figure S1**. To optimize the cross-section weightings, the weightings were initially set to the YL tabulated cross-section values, with an estimate for the Ti 4*p* calculated by multiplying the Ti 4*s* by the Ge 4*p*/4*s* ratio, which gave the lowest $\boldsymbol{RSS}$ compared to the other 'non-optimized' methods. The weightings were allowed to vary freely to achieve the best fit for both the anatase and rutile polymorphs. The average was then taken from the two phases to generate an optimised value for both phases. The individually optimised values and the final values applied are listed in **Table 2**. The O*s* contribution was rounded down to 0.5 as this reduced the $\boldsymbol{RSS}$ value for rutile, without affecting the $\boldsymbol{RSS}$ value for anatase.

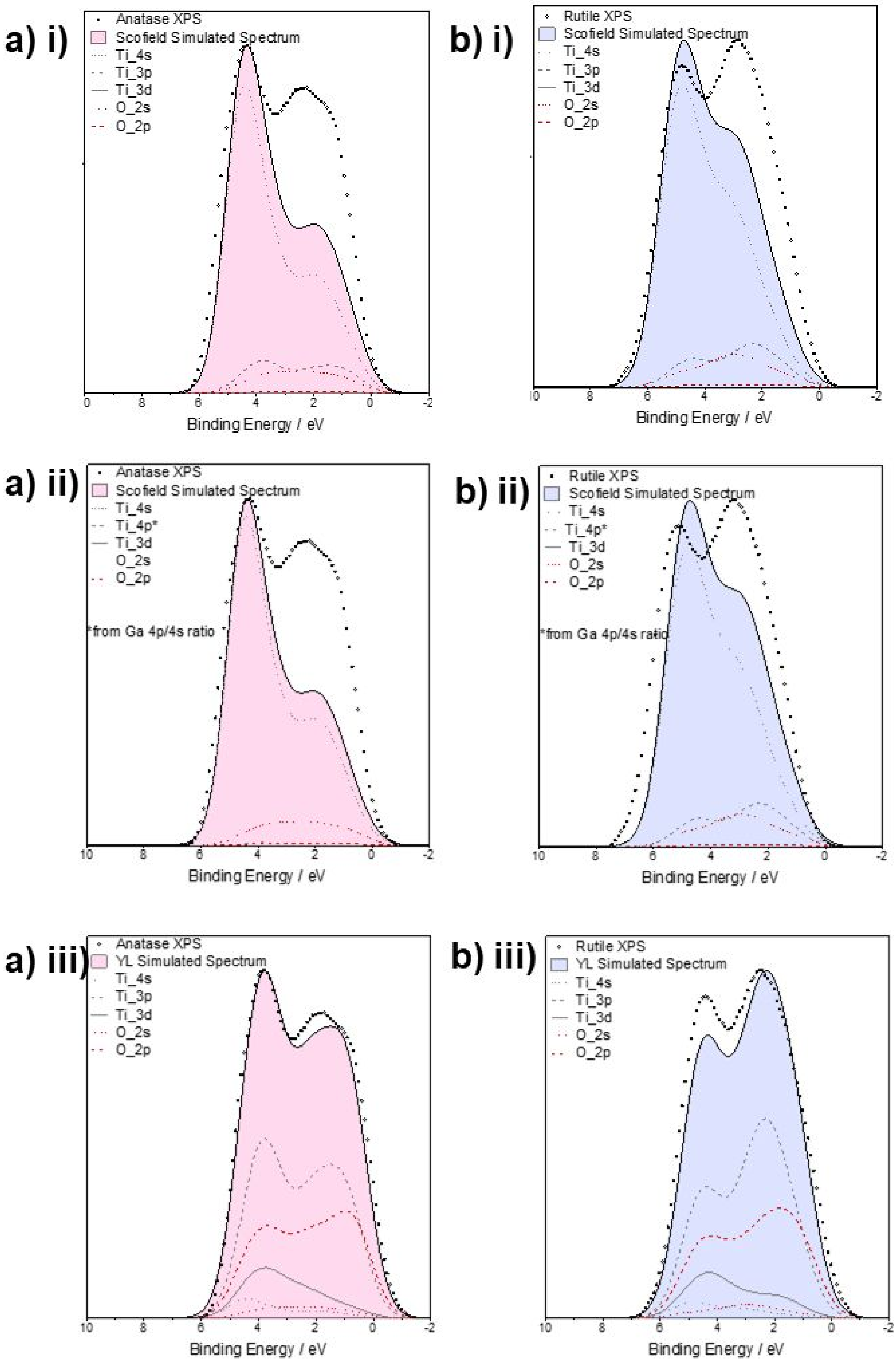
a) i)
Anatase XPS
Scofield Simulated Spectrum
Ti_4s
Ti_3p
Ti_3d
O_2s
O_2p
Binding Energy / eV
b) i)
Rutile XPS
Scofield Simulated Spectrum
Ti_4s
Ti_3p
Ti_3d
O_2s
O_2p
Binding Energy / eV
a) ii)
Anatase XPS
Scofield Simulated Spectrum
Ti_4s
Ti_4p*
Ti_3d
O_2s
O_2p
*from Ga 4p/4s ratio
Binding Energy / eV
b) ii)
Rutile XPS
Scofield Simulated Spectrum
Ti_4s
Ti_4p*
Ti_3d
O_2s
O_2p
*from Ga 4p/4s ratio
Binding Energy / eV
a) iii)
Anatase XPS
YL Simulated Spectrum
Ti_4s
Ti_3p
Ti_3d
O_2s
O_2p
Binding Energy / eV
b) iii)
Rutile XPS
YL Simulated Spectrum
Ti_4s
Ti_3p
Ti_3d
O_2s
O_2p
Binding Energy / eV

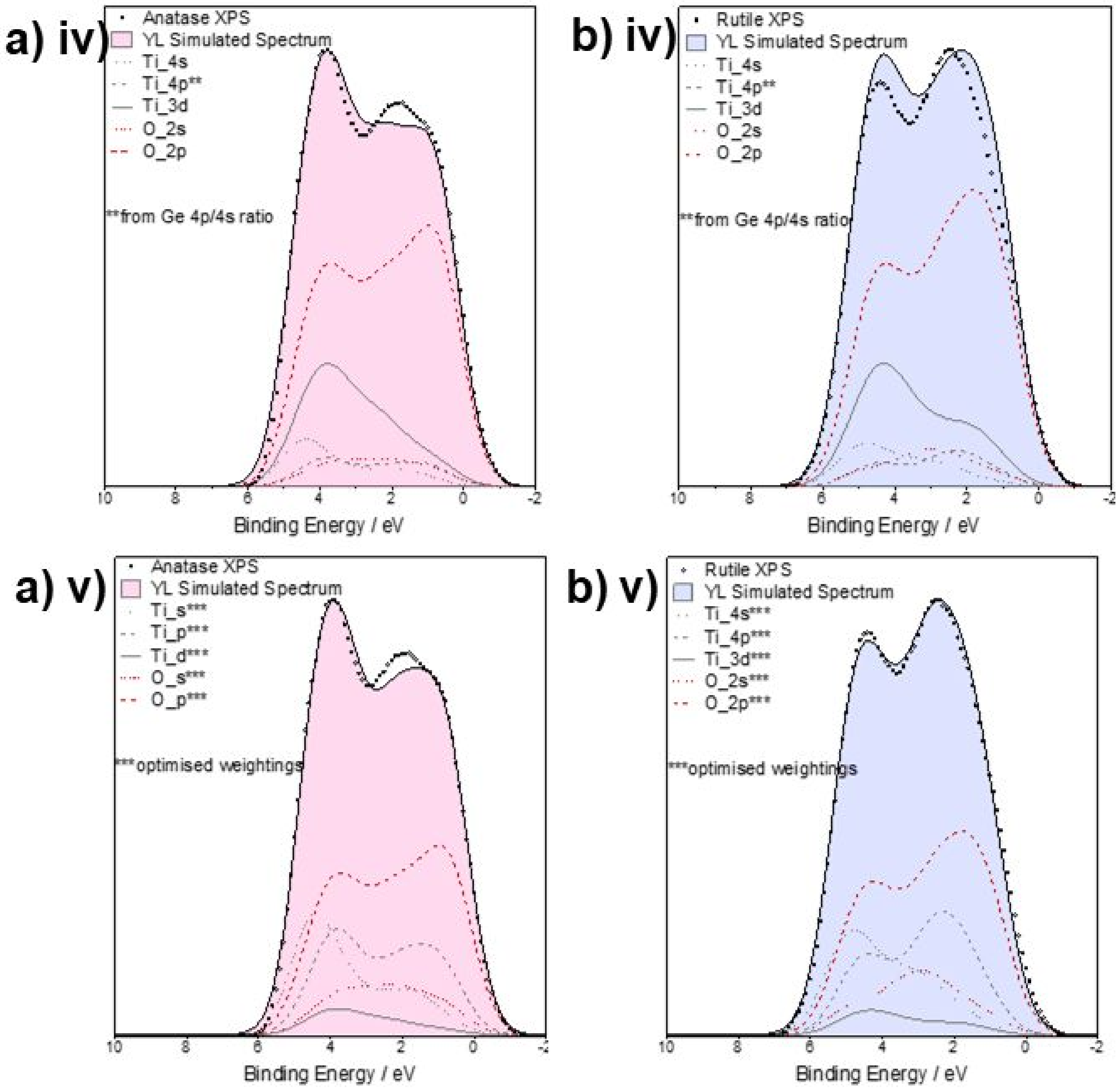

Figure S1. Simulated spectra resulting from different methods of photoionization cross-section correction for **a)** anatase and **b)** rutile. The different methods trialled are labelled **i)** to **v)** and correspond to the methods and ***RSS*** values listed in Table 3.

Table S1. Cross-section values applied during refinement.

**YL cross-section**		**Scofield cross-section**	
O 2s	9.50×10^{-4}	*O 2s*	1.42×10^{-6}
O 2p	6.00×10^{-5}	*O 2p*	7.75×10^{-10}
Ti 4s	2.50×10^{-4}	*Ti 4s*	2.98×10^{-6}
Ti 3d	8.50×10^{-5}	*Ti 3d*	6.70×10^{-11}
Ti 3p	1.83×10^{-3}	*Ti 3p*	2.55×10^{-7}
*Ti 4p**	1.23×10^{-4}	*Ti 4p***	2.03×10^{-8}

*from *Ge 4p/4s* ratio **from *Ga 4p/4s* ratio

Table S2. Optimised cross-section weightings calculated by fitting the simulated spectra with experimental spectra for the anatase and rutile polymorphs. The *Ti 4p** has been estimated by multiplying the *Ti 4s* by the *Ge 4p/4s* ratio.

Orbital	Initial values (YL)[1]	Anatase	Rutile	Average weighting	Normalised weighting	Weightings used	Optimized cross-section
O 2s	9.50×10^{-4}	1.00	1.00	1.00	0.57	0.5	4.75×10^{-4}
O 2p	6.00×10^{-5}	0.23	0.47	0.35	0.20	0.2	1.20×10^{-5}
Ti 4s	2.50×10^{-4}	0.98	1.32	1.15	0.65	0.7	1.75×10^{-4}
Ti 4p	1.23×10^{-4}	1.36	2.16	1.76	1.00	1	1.23×10^{-4}
Ti 3d	8.50×10^{-5}	0.00	0.20	0.10	0.06	0.06	5.10×10^{-6}

Table S3. Comparison of the ***RSS*** from fitting the experimental anatase and rutile VB spectra with simulated spectra using different methods of cross-section correction.

Correction method	RSS Anatase	RSS rutile
***i)** Scofield (Ti 3p)*	4.06	2.13
***ii)** Scofield (Ti 4p)*	4.47	2.81
***ii)** YL (Ti 3p)*	0.13	0.34
***iv)** YL (Ti 4p)*	0.06	0.20
***v)** Optimised**	0.03	0.04

*from Table 2.

Comparison of DFT and empirical fitting models

The DFT fitting model was compared to an empirical fitting model, using anatase and rutile XPS VB spectra measured from the single-phase films (**Figure 1** main text). These models were fitted to VB spectra recorded from a 12 x 12 grid of points across mixed-phase r-corner and r-centre films, each point around 2 mm apart. During fitting, the binding energy positions of each component were allowed to vary freely, their intensity ratios were allowed to vary between 0 and 100 %, and a scale factor was applied such that the model total was equal to the maximum intensity of the experimental spectra. The anatase:rutile ratio with the minimum RSS was used to estimate the surface phase fraction at that point.

The resulting fits from applying the models to 5 points on the r-centre sample are shown in **Figure S2**. The same trend of increasing anatase composition was observed for the selected points in the r-centre sample for both the empirical and DFT-simulated models, with excellent agreement seen for the estimated phase fraction of anatase for all points across both samples (**Figure S3**). The DFT-simulated fitting model tends to underestimate the phase fraction of anatase compared to the empirical fitting model. One potential cause is the disagreement between the experimental and simulated spectrum of anatase in the interpeak region (**Figure 1** main text). This could be due to the higher reactivity of the anatase phase leading to more adventitious molecules adsorbed on the surface that may contribute to the experimental VB spectrum.

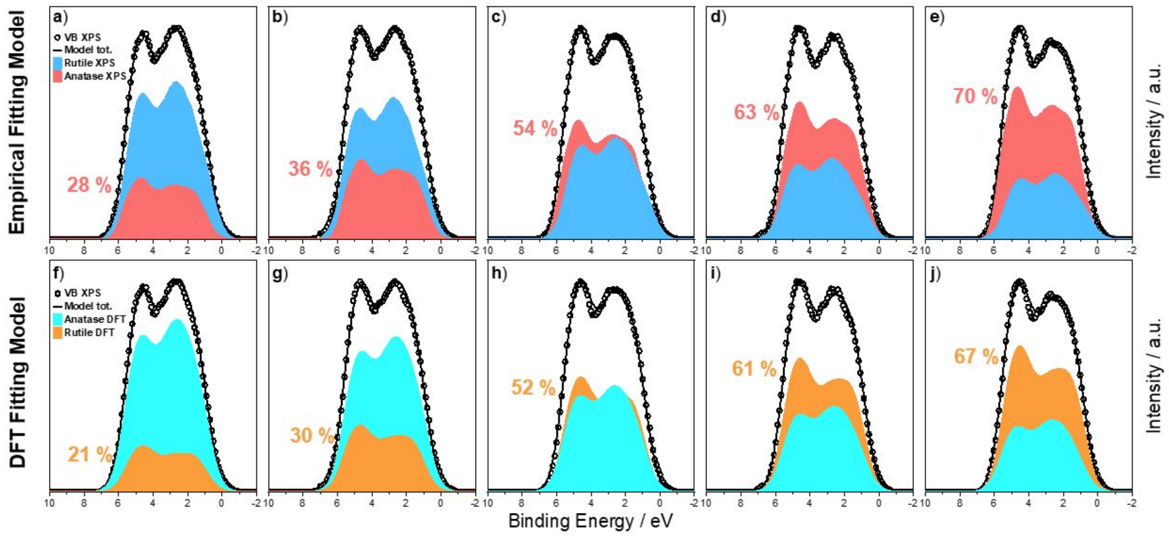

Figure S2. Mixed-phase fitting models based on empirical fitting with XPS spectra **(a-e)** and fitting with DFT-simulated spectra **(f-j)**, applied to VB XPS spectra taken from 5 points of mixed-phase composition from the r-centre sample. Each graph depicts the ratio of each phase that generates the lowest residual sum of squares ($\boldsymbol{RSS}$) and is labelled with the corresponding phase fraction of anatase.

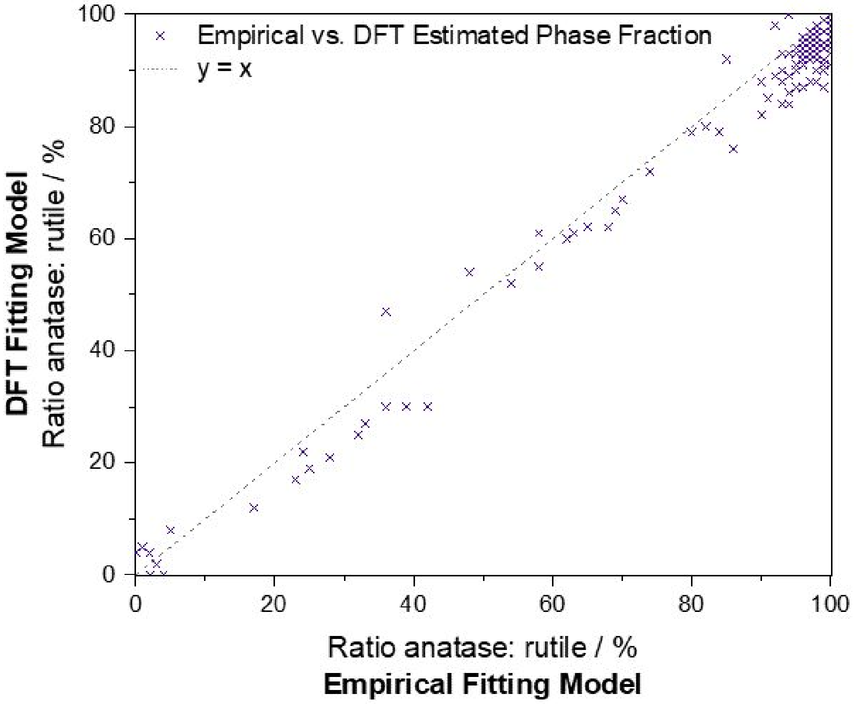

Figure S3. Comparison of the estimated surface phase fraction of anatase from fitting VB XPS spectra recorded on mixed-phase TiO_2 using an empirical fitting model and a DFT-simulated fitting model.

Raman Spectroscopy Composition Mapping Method

Raman spectroscopy was applied to investigate the composition across the mixed-phase TiO_2 films along selected cross-sections of each sample. Phase quantification was achieved using a methodology described elsewhere.[2] The analysis region was confined to 310–750 cm^{-1}, with anatase modes[3] at 395 cm^{-1} (B_{1g}), 515 cm^{-1} (A_{1g}+B_{1g}) and 637 cm^{-1} (E_g) and rutile modes[4] at 438 cm^{-1} (E_g) and 605 cm^{-1} (A_{1g}), respectively. The spectra were normalised and fitted with linear background functions before the fitting of Raman bands using Lorentzian functions. The frequencies of the individual modes were allowed to vary ± 5 cm^{-1} and the FWHM was limited to the average values recorded over 10 points on the single-phase anatase and rutile thin films. Examples of the deconvoluted Raman spectra for three points on the r-centre sample are shown in **Figure S4.** The relative intensities of the fitted Lorentzian components were used to estimate the compositions at each point, which are indicated in the figure. Comparison between composition cross-sections estimated from XPS and Raman analysis are shown in **Figure S5**. From the Raman analysis, the reference samples were determined to be 100 % anatase and rutile, and the annealed areas in the r-centre and r-corner samples were found to contain regions of up to 89 % and 96 % rutile, respectively. The film quality was reduced at the edges of the samples, which is the probable cause for disagreement at these points. The discrepancies away from the edges arise from the different probing depth of the two techniques.

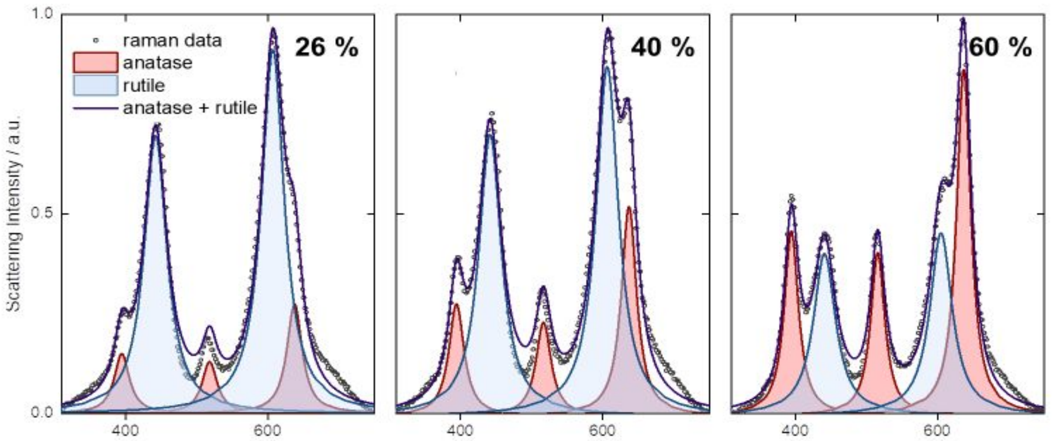

Figure S4. Mixed-phase Raman spectroscopy models fitted to three points on the r-centre film. The corresponding estimated anatase content are shown in the top right of each plot.

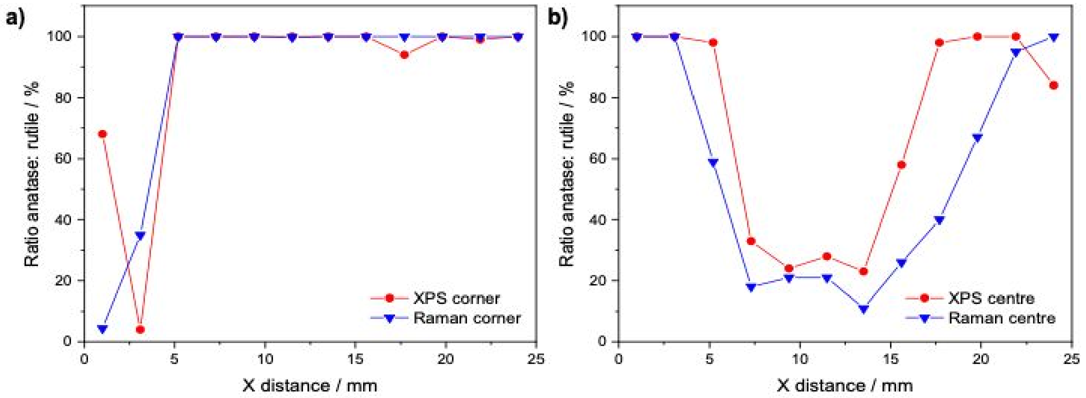

Figure S5. Estimated compositions from applying the mixed-phase Raman models to cross-sections through the mixed phase films **(a)** r-corner and **(b)** r-centre. The corresponding compositions from XPS analysis are shown in each figure.

Photocatalytic Activity Mapping Method

The photocatalytic properties of the TiO2 films were probed using a standard test, based on the photoreduction of resazurin dye (*smart* ink).[5] The underlying mechanism of the process **Figure S6a**)[6] relies on the photo-oxidation of a sacrificial electron donor (SED) driven by positive holes (h^+) trapped at the photocatalyst surface upon light excitation. The SED used in this formulation is typically glycerol. In turn, the resulting electron-rich surface can irreversibly reduce the resazurin molecule (R_z) to resorufin (R_f), which is evidenced by a colour change from blue to pink. This transformation can be easily monitored visually and by photographic analysis. Selected photographs from the *smart* ink test monitoring the performance of pure and mixed-phase anatase and rutile films are shown in **Figure S6b**. The transformation of R_z (blue) into R_f (pink) was evidenced within the first minutes of irradiation for most films except for pure rutile, which is inactive in this process. Prolonged irradiation (t= 100 min) lead to the full degradation of the ink.

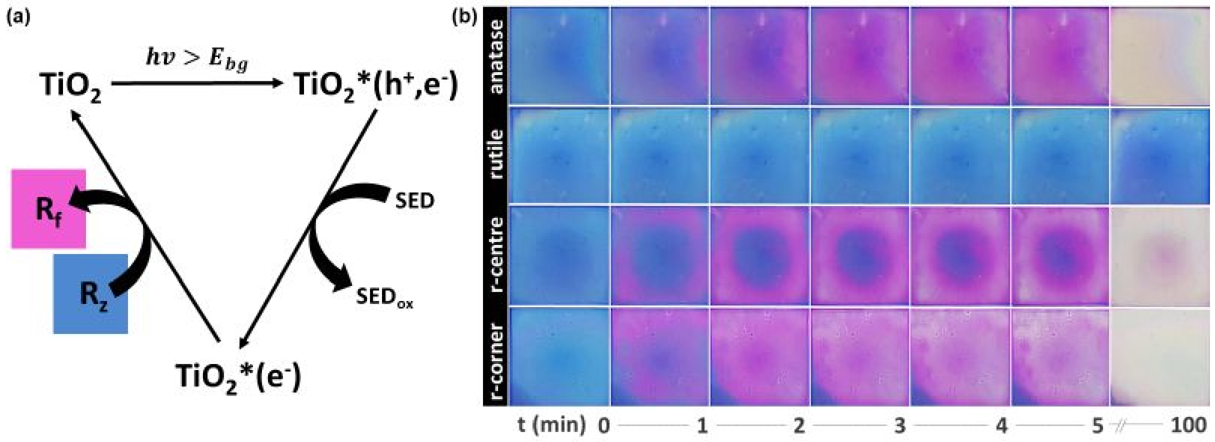

Figure S6. (**a**) Reaction scheme proposed for the transformation of the resazurin-based ink upon irradiation.[6] In our case, glycerol acted as sacrificial electron donor (SED) in the ink formulation. The scavenging of holes (h^+) from the UV-excited semiconductor results in an electron-rich surface that reduces the resazurin dye (Rz) to resorufin (Rf), with an associated colour change from blue to pink. **(b)** Photographic monitoring of the reaction at selected time intervals during UV exposure (λ= 365 nm, *I*= 2 mW cm^{-2}).

To further quantify these transformations, colour mapping of the photographs was carried out averaging RGB values across a 12×12 grid for the mixed phase samples for direct comparison with XPS mapping. Following the procedure described in the ISO test,[5] the RGB values were transformed into normalised *RGB(red)* component, R_t, at irradiation time *t*, according to **Eq. S2**.

$$R_t = \frac{RGB(R)_t}{RGB(R)_t + RGB(B)_t + RGB(G)_t} \qquad \textbf{Eq.S2}$$

Where $RGB(R,B,G)_t$ correspond to red, blue and green components, respectively.

Figure S7 illustrates an example of this analysis after t= 100 s irradiation. Following the evolution of R_t values as a function of irradiation time across the entire 12×12 grid in the samples allowed for an estimation of dye photoreduction rates, k_{Rt}. Examples of R_t curves are plotted in **Figure S5**. k_{Rt} values were estimated from linear regression of the initial steps in these curves. To account for photon-energy gradients during the irradiation experiments, measured in the range of 1.8−2 mW cm^{-2}, formal quantum efficiencies, ξ (units, s^{-1} photon^{-1}), were then estimated from k_{Rt} values per incident photon, for a fair comparison among the samples (**Figure 5**). For simplicity, these calculations account for photons at λ= 365 nm (maximum of light emission band) only, since the UVX detector we used to measure irradiance has a broad detection sensitivity across 300-400 nm and the lamp has a broad emission spectrum within the same range. Nevertheless, this assumption affects all the samples equally and accurate comparison can still be established, albeit absolute ξ values will likely be underestimated.

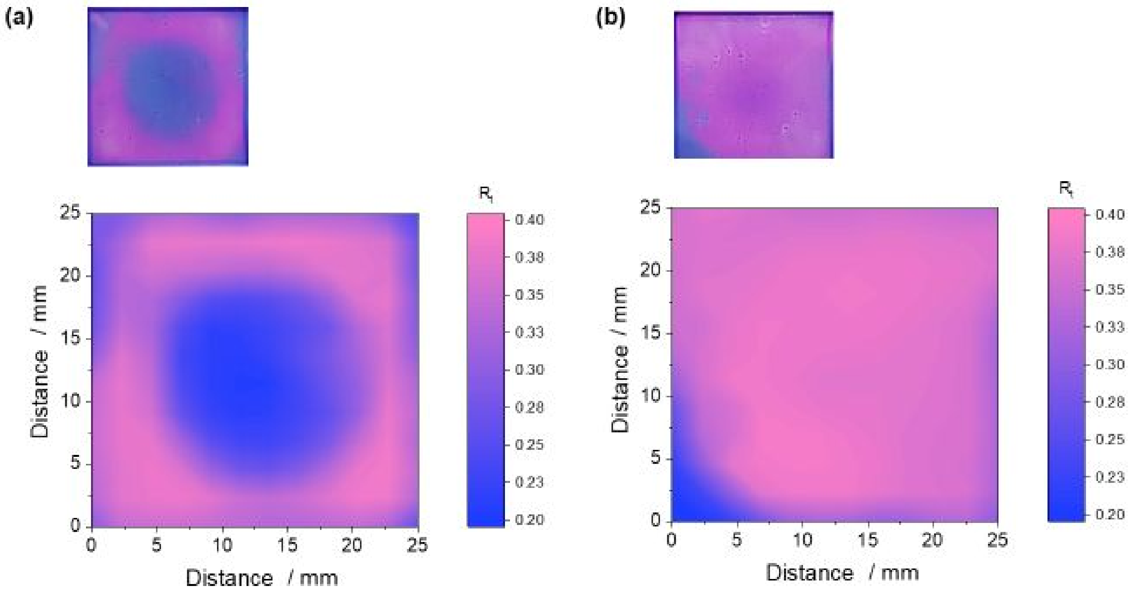

Figure S7. RGB mapping based on normalised red component, Rt (**eq. S1**) of **(a)** r-centre and **(b)** r-corner samples after t= 100 s irradiation under UV light. The pink and blue areas in the analysis correspond to anatase- and rutile-rich regions, respectively. The corresponding photographs of the samples are included for reference.

REFERENCES

1. Yeh, J. J.; Lindau, I., Atomic subshell photoionization cross sections and asymmetry parameters: $1 \leqslant Z \leqslant 103$. *Atomic Data and Nuclear Data Tables* **1985,** *32* (1), 1-155.
2. Zanatta, A. R., A fast-reliable methodology to estimate the concentration of rutile or anatase phases of TiO2. *AIP Advances* **2017,** *7* (7), 075201.
3. Ohsaka, T.; Izumi, F.; Fujiki, Y., Raman spectrum of anatase, TiO2. *Journal of Raman Spectroscopy* **1978,** *7* (6), 321-324.
4. Porto, S. P. S.; Fleury, P. A.; Damen, T. C., Raman Spectra of TiO2, MgF2, ZnF2, FeF2, and MnF2. *Physical Review* **1967,** *154* (2), 522-526.
5. ISO 21066:2018 Fine ceramics (advanced ceramics, advanced technical ceramics) - Qualitative and semiquantitative assessment of the photocatalytic activities of surfaces by the reduction of resazurin in a deposited ink film. https://www.iso.org/standard/69815.html.
6. Mills, A.; Wang, J.; Lee, S.; Simonsen, M., An intelligence ink for photocatalytic films. *Chemical Communications* **2005,** (21), 2721-2723.
